# How memory effects, check dams, and channel geometry control erosion and deposition by debris flows

**DOI:** 10.1038/s41598-020-71016-8

**Published:** 2020-08-20

**Authors:** T. de Haas, W. Nijland, S. M. de Jong, B. W. McArdell

**Affiliations:** 1 Department of Physical Geography, Universiteit Utrecht, Utrecht, The Netherlands; 2 Swiss Federal Institute for Forest, Snow and Landscape Research WSL, Birmensdorf, Switzerland

**Keywords:** Natural hazards, Geomorphology

## Abstract

Debris flows can grow greatly in size and hazardous potential by eroding bed and bank material, but effective hazard assessment and mitigation is currently hampered by limited understanding of erosion and deposition dynamics. We have collected high-resolution pre- and post-flow topography for 6 debris flows over a 3 km long unconsolidated reach of the Illgraben channel in the Swiss Alps with drone-based photogrammetry. We show that the spatio-temporal patterns of erosion and deposition in debris-flow torrents are highly variable and dynamic. Check dams strongly control the spatial patterns of erosion and deposition. We identify a memory effect where erosion is strong at locations of strong deposition during previous flows and vice versa. Large sediment inputs from subcatchments initially result in new channel erosion through the subcatchment deposits and simultaneous upstream deposition, likely as a result of backwater effects. It is generally believed that erosion increases with debris-flow magnitude, but we show that there is a limit to debris-flow bulking set by channel geometry. These findings provide key guidelines for flow volume forecasting, emphasizing the importance of memory effects and the need to resolve both erosion and deposition in predictive models.

## Introduction

Debris flows are rapid destructive masses of soil, rock and water that are common natural hazards in mountainous regions worldwide^1–3^. The continued expansion of human populations into mountainous regions has greatly increased the risk associated with debris flows^4^, and hazards are expected to increase as a result of global climate change^5–7^. Debris flows are typically generated by small landslides or runoff in mountain catchments, after which they flow down onto (inhabited) alluvial fans and valley floors^8^. They may entrain large amounts of bed sediment when rushing down mountainsides, possibly increasing in size by several orders of magnitude^9–14^. Estimation of flow volumes is critical for assessment of flow hazard and design of mitigation measures, because flow volume is the prime control on flow velocity, peak discharge, inundation area^15–17^ and the number of fatalities^4^. Unfortunately, limited understanding of debris-flow erosion and deposition dynamics currently hampers debris-flow volume estimation and hazard assessment and mitigation.

Resolving debris-flow deposition is often ambiguous in numerical models^18–22^, and few models have been explicitly tested for their performance in describing deposition due to debris flows. There has been a recent increase in the number of numerical models incorporating erosion^21–23^, but the inconsistency in erosion rate equations as a result of a lack of a unified theory still results in a disparity of model outcomes. Much of our understanding of debris-flow erosion stems from theoretical considerations^24^ and physical scale experiments^13,25–28^, while there is a relative scarcity of field data^11,12,29–32^ as a result of the infrequent nature of debris flows, the rough terrain in which they occur, and the high time and cost demands of field measurements. Analysis of field data is often hampered by unknown boundary conditions and material properties^11,12^, and is often based on local point or cross-section measurements^12,31^, single time-steps^32^, and measurements are typically only available for small areas^29,30^. Debris-flow torrents are often complex systems, with spatially variable channel geometries and bed materials, sediment contributing subcatchments, and in inhabited areas numerous check dams which locally stabilize the channel. To extrapolate findings from theoretical considerations and physical-scale experiments to the complex field environment and to perform accurate flow-volume estimates for hazard assessment and mitigation, we need to develop a detailed understanding of the largely unknown spatio-temporal erosion–deposition patterns and processes, and associated flow bulking in debris-flow torrents.

Here we present a drone-based time- and cost-effective method for monitoring topographic changes over long reaches of debris-flow channels. We present erosion and deposition patterns in the Illgraben torrent in the Swiss Alps as a result of six debris flows and subcatchment activity over a 3.3 km long unconsolidated reach with check dams, and compare these erosion and deposition patterns with in-situ flow measurements. Our work shows that erosion and deposition patterns can be highly heterogeneous, and sheds light on four crucial but generally overlooked aspects that control erosion and deposition in debris-flow torrents: (1) check dams; (2) memory effects; (3) effects of subcatchment inputs; (4) and channel geometry.

## Illgraben catchment and torrent

**Figure 1:**
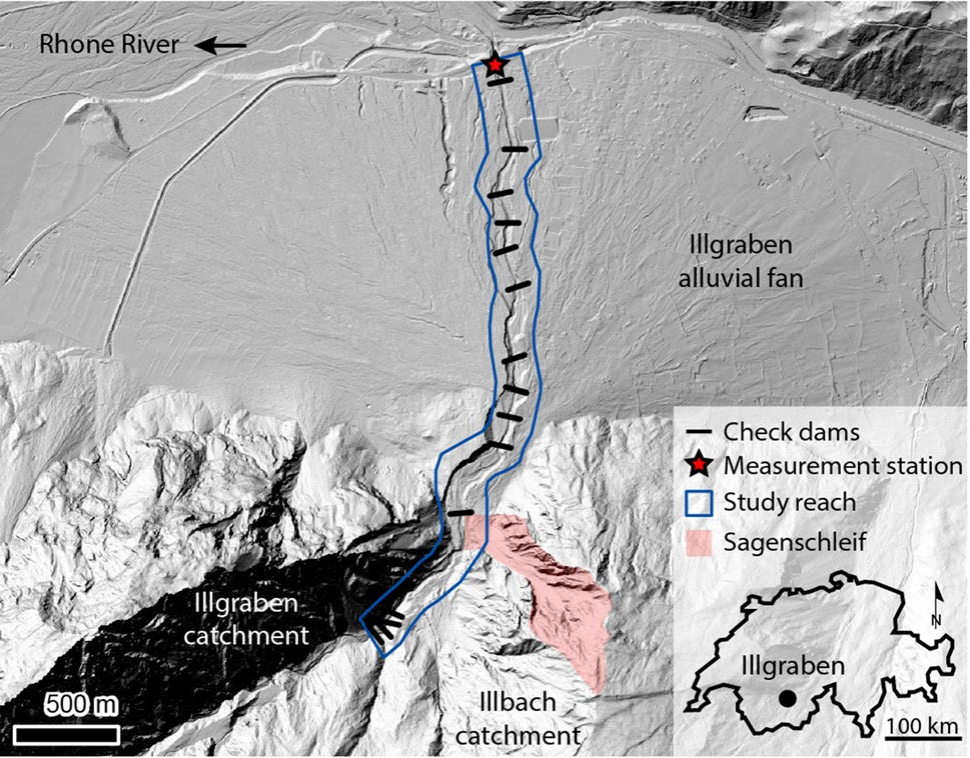
Overview of the Illgraben catchment, fan, and channel, and the Sagenschleif subcatchment. The blue outline corresponds to the area for which we derived drone-based elevation models. The black lines indicate the location of the check dams that actively control the channel bed, and correspond to the check dams shown in Fig. 2. Fan apex is located at 46°17′37.76″N, 7°38′1.15″E. North is up.

The Illgraben catchment in the Swiss Alps (Fig. 1) has a long history of debris flows^33,34^, and an extremely high debris-flow frequency of approximately 5 debris flows and debris floods per year since 2000, annually transporting ~ 100,000 m^3^ of debris to the Rhone River^35^. The catchment extends from the summit of the Illhorn mountain (elevation 2,716 m a.s.l.) to the Rhone River on the valley floor (610 m a.s.l.)^36^. The catchment has a total area of 8.9 km^2^, but all debris flows originate from a 4.6 km^2^ subcatchment. This subcatchment is composed of massive dolomites on its northwest wall and layers of quartzite, conglomerates, and calcareous sedimentary rocks on its southeast valley wall^37^. Debris flows are generally triggered by intense rainfall during summer storms between May and October.

The channel stretching from the Illhorn mountain to the Rhone River has a length of ~ 6.5 km, of which the distal 4.8 km hosts 28 check dams which cause vertical drops of several meters along the channel bed. The reach with check dams has an unconsolidated bed^29^. For the most downstream 2 km the channel traverses a large alluvial fan. Just upstream of the point where the channel leaves the catchment a small (0.1 km^2^) but recently very active subcatchment is present, the Sagenschleif, which currently contributes large quantities of sediment to the channel.

A few tens of meters upstream of the confluence with the Rhone River an automated observation station is operated by the Swiss Federal Institute for Forest Snow and Landscape Research (WSL), which records flow-front velocity, flow depth, and normal and shear forces, and collects imagery^36,37^. From these observations, it is possible to calculate the front discharge of a debris flow and to estimate its volume^38^.

## Channel topography measurements

We have measured before and after channel-bed topography of the downstream 3.3 km of the channel through drone-based photogrammetry for six debris-flow events that occurred between December 2018 and July 2019 (Table 1). These flows had maximum flow depths ranging from 0.6 to 2.6 m, flow front velocities ranging from 0.9 to 5.6 m/s, front discharges ranging from 4 to 122 m^3^/s, and volumes ranging from 3,000 to 75,000 m^3^, at the observation station (Table 1; Suppl. Figure 1). Channel bed topography was measured on 8 November 2018, and 29 April, 16 June, 22 June, and 4 July in 2019 (Suppl. Table 1). Thereby, we capture the erosion and deposition patterns caused by the debris flow of December 2018, the cumulative effect of the two flows on 10 June, the flow of 21 June, and the cumulative effect of the two flows of early July in 2019. In the winter of 2018/2019 large quantities of sediment were transported into the Illgraben channel from the Sagenschleif tributary, mainly by debris flows (Fig. 1).

**Table 1:** Flow characteristics of the studied debris flows measured at the observation station.

Date	Max stage (m)	Frontal velocity (m)	Front discharge (m^3^/s)	Volume (m^3^)
December 4, 2018	~ 0.7	–	–	–
June 10, 2019	0.7	0.9	4	3,000
June 10, 2019	0.6	2.4	8	20,000
June 21, 2019	2.6	5.6	122	35,000
July 2, 2019	1.7	3.8	45	75,000
July 3, 2019	0.7	1.1	6	39,000

Event volumes and front discharge were estimated using the methods described in^38^. The detailed flow hydrographs are shown in Suppl. Fig. 1. The station was under maintenance on December 4, 2018, and therefore most flow properties are unknown.

## Spatio-temporal patterns of erosion and deposition

**Figure 2:**
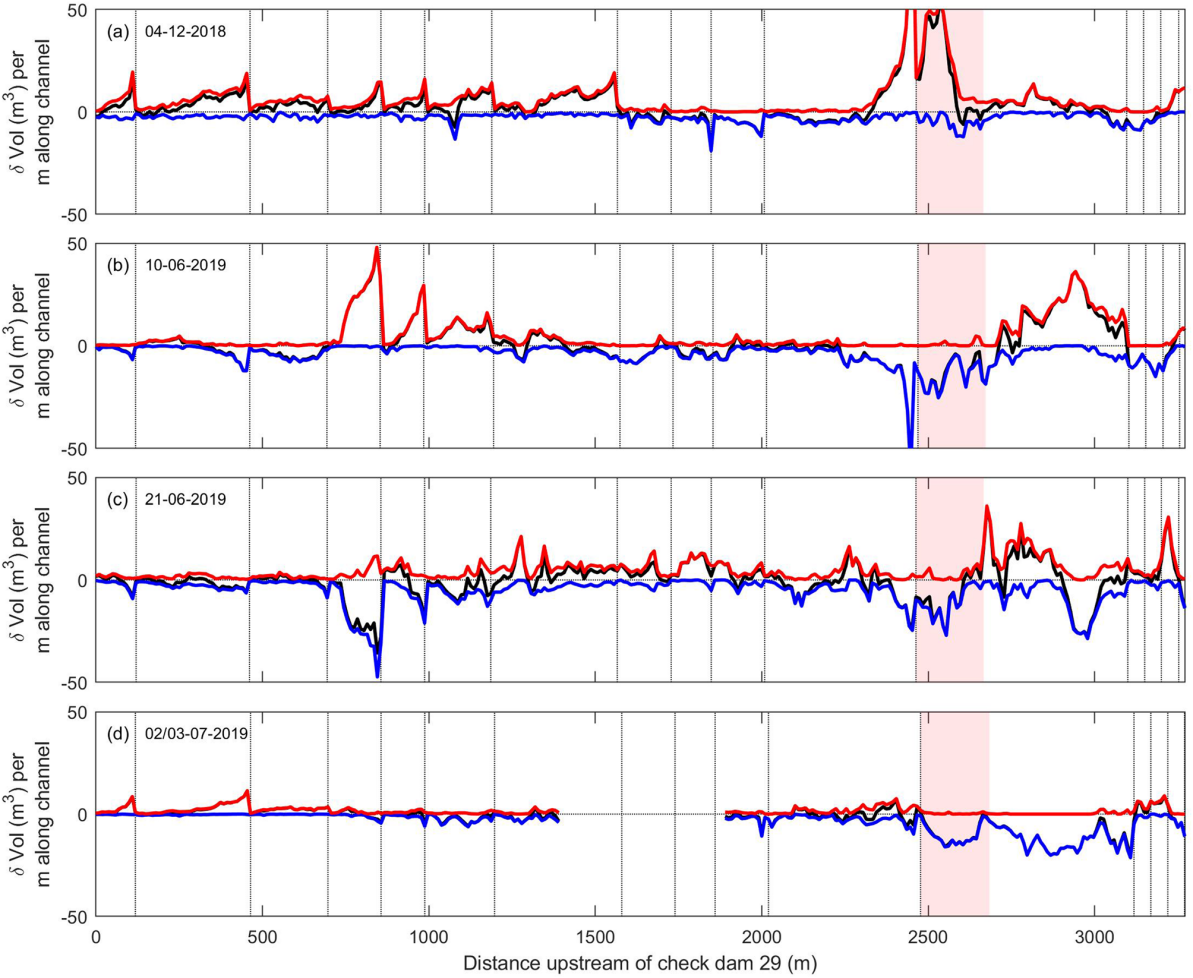
Spatio-temporal patterns of erosion and deposition. The red, blue, and black lines indicate the volumes of deposition, erosion, and the net volume change per meter along channel, respectively. The dashed lines indicate the locations of the check dams that affect the channel. The red fill indicates the location of the Sagenschleif subcatchment (Fig. 1).

**Figure 3:**
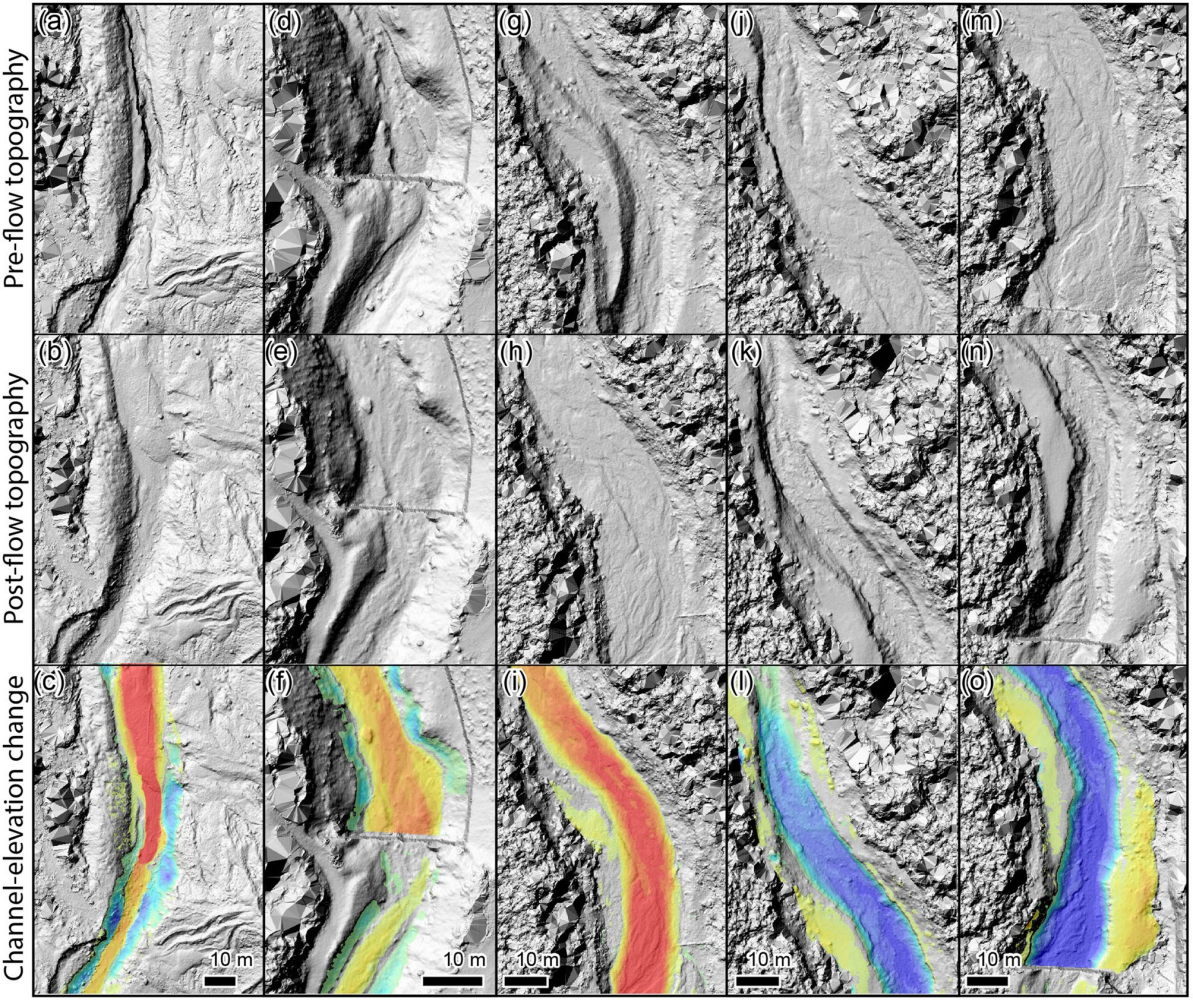
Examples of erosion and deposition patterns. (**a**–**c**) Pre-flow topography, post-flow topography, channel-elevation change showing how the Sagenschleif activity buried the channel (distance upstream of check dam 29 (x = 2600–2700 m in Fig. 2a). (**d**–**f**) Deposition downstream of a check dam by the 4 December 2018 flow (x = 420–500 m in Fig. 2a). (**g**–**i**) Extensive deposition by the two debris flows on 10 June 2019, fully burying the initial channel (x = 760–850 m in Fig. 2b). (**j**–**l**) Strong erosion and the formation of a new channel by the June 21 debris flow, removing deposits from the June 10 flows (x = 730–810 m in Fig. 2c). (**m**–**o**) Simultaneous channel thalweg erosion and bank deposition during the large flow of June 21, generally leading to net deposition (x = 780–860 m in Fig. 2c). Orientation of all images is to the north, and flow is from bottom to top. Panels show hillshaded DEMs, for the channel-elevation change panels overlain by the DEM of difference, on which warm colours denote deposition and cold colours denote erosion. Value range from red to blue is − 3 to 3 m channel-bed elevation change, values ranging between − 0.25 and 0.25 m are transparent. See Suppl. Movies 1–4 for wider context change over all time steps.

The topographic changes within the Illgraben channel reveal a strong spatio-temporal heterogeneity in erosion and deposition patterns, which are also strongly affected by the check dams (Fig. 2). The flow from December 2018 shows a sharp transition from net erosion to net deposition around 1,600 m upstream of check dam 29 (Fig. 2a). The check dams cause a strong saw-tooth pattern of deposition, with most deposition just downstream of the check dams after which deposition gradually decreases in the downstream direction towards approximately zero at the crest of the next check dam (Fig. 3d–f; Suppl. Movie 2). Typical cross-channel deposition volumes are roughly similar in the reaches between the check dams. Mean channel depth has decreased by up to 1 m downstream of the check dams, but maximum deposition in the middle of the thalweg typically exceeds 2 m. The patterns of net erosion above 1,600 m upstream of check dam 29 shows an inverse, but otherwise similar, spatial pattern, with maximum erosion downstream of the check dams. The geomorphic activity from the Sagenschleif has delivered large volumes of sediment into the Illgraben channel below and slightly downstream of the subcatchment totally filling the channel and locally raising mean bed level by up to 5 m (Fig. 3a–c; Suppl. Movie 1).

The two debris flows on June 10 have resulted in alternating net erosion and net deposition along the channel (Fig. 2b). A new channel has been cut through the Sagenschleif deposits in the Illgraben channel resulting in net erosion, but at the same time there was deposition in the 500 m upstream of the Sagenschleif, likely as a result of backwater induced by deposition at the Sagenschleif. Further downstream the June 10 debris flows have primarily caused net erosion, apart from the ~ 500 m reach between 700 and 1,200 m upstream of check dam 29. In this reach unusually large quantities of sediment have been deposited, locally raising mean bed level > 2 m (Fig. 3 g–I; Suppl. Movie 3).

The large flow of June 21 (Suppl. Figure 1) continued eroding the Sagenschleif deposits, and was also associated with sediment deposition upstream of the Sagenschleif (Fig. 2c). Interestingly, most deposition took place directly upstream of the Sagenschleif, while further upstream, where maximum deposition took place during the previous flows, there is now net erosion (~ 2,900 m upstream of check dam 29). Downstream of the Sagenschleif there is a reach of > 1,000 m over which substantial erosion and deposition simultaneously took place. This was the result of the large size of this flow, resulting in thalweg erosion and simultaneous deposition by the formation of levees and other deposits on top of overbanks (Fig. 3j–o; Suppl. Movie 4). Over the majority of this reach this resulted in net deposition. Further downstream, around a distance of 1,200 m upstream of check dam 29 there is a shift to net erosion. Along this reach there is a striking correspondence between the location of erosion of the flow of June 21 and the locations of deposition during the previous flows. Large volumes have been eroded by the June 21 debris flows between 700 and 1,200 m upstream of the confluence, which is exactly the location of extensive deposition by the two flows on June 10. From 450 to 700 m there is a sudden transition to little erosion nor deposition—in this reach erosion and deposition during the previous flows balances out. Downstream of 450 m upstream of the confluence there is net erosion with a similar pattern as during the two previous flows on June 10. Over this reach the combined erosion of the 10 and 21 June flows seems to counterbalance the deposition that occurred during the December 2018 flow.

The flows of June 2 and 3, of which the flow of June 2 was of considerable size, caused relatively minor channel bed changes (Fig. 2d). These flows further eroded the Sagenschleif deposits, and also removed the backwater deposits that formed behind the Sagenschleif during the previous flows. Directly downstream of the Sagenschleif there was limited mixed erosion and deposition, shifting to net erosion around 2000 m upstream of check dam 29. Net deposition occurred downstream of 700 m upstream of check dam 29. Deposition here seems to counterbalance the erosion of the previous 3 flows that were net erosive in this reach.

## Effects of check dams on erosion and deposition patterns

In the European Alps and many other mountainous areas, most debris-flow channels that pose a threat to communities and infrastructure have check dams. Check dams are generally applied to debris-flow channels to prevent vertical and lateral channel erosion and movement^39–41^. In the Illgraben channel the check dam surfaces are in line with the bed surface, and act as bed sills that provide a locally fixed base level. As such, the check dams exert a strong control on the spatio-temporal erosion and deposition trends observed in the Illgraben channel (Figs. 2, 3), with the most pronounced changes generally occurring directly downstream of a check dam and moving to approximately zero at the downstream check dam. Furthermore, in the study reach bed erosion was more pronounced than bank erosion, which may be the result of the check dams preventing lateral channel migration and stabilizing banks.

Severe scour has often been identified downstream of check dams, providing an engineering and safety problem^39^. Our data shows that deposition may also be very large downstream of a check dam, possibly buffering the total amount of scour over a number of events. Because of the fixed bed level at check dams, erosion automatically implies a reduction in the effective slope of the channel within the check dams. Such as reduction in channel slope then increasing the likelihood of deposition in a subsequent flow and vice versa. In contrast, erosion in unconsolidated channels without check dams lowers the channel bed more uniformly, and will thus lead to far less or no channel slope reduction. This suggests that torrents with check dams may be more prone to memory effects.

## Memory effect controls on erosion and deposition patterns

Previously many flow parameters including flow volume^42^, flow depth^29,31^, flow velocity^43,44^, discharge^45^, bed slope^12,46^, shear stress^29,31,47^, grain collisional stress^48–50^ and bed wetness^25^, have been shown to positively correlate with erosion rate. Although we do not doubt that these parameters do indeed affect erosion and deposition by debris flows, our results do suggest that memory effects may play a similarly important role in nature.

Our observations show that erosion and deposition in a debris flow strongly depend on the erosion and deposition by previous debris flows and subcatchment activity. Localities where substantial deposition occurred have a high probability of substantial erosion during subsequent flow(s) and vice versa. This might be the result of the channel-bed deviation around an equilibrium profile, partly affected by check dams as explained above. Alternatively, for some of the localities where we observe a memory effect, namely removal of deposits by the 21 June 2019 debris flow, the observed patterns might result from the short residence time of 11 days between this and the previous flow. Therefore, the removed deposits may still have had an elevated pore-water content and were easily eroded^25^. Still, our observations show that the channel carved by the 21 June 2019 flow does differ from that present before the 10 June 2019 flows. In addition, the 2 and 3 July flows did not substantially erode or deposit despite their relatively large flow depth and velocity, likely as a result of the already strongly scoured thalweg by the very large previous flow of June 21.

Furthermore, large sediment input from subcatchments that block a channel affect erosion and deposition patterns during multiple subsequent flows. We find net erosion through these deposits as a new channel is carved, but at same time upstream deposition as a result of backwater. In this particular example the backwater deposits are removed after a few flows, likely because around that time an efficient debris-conveyance corridor through the subcatchments deposits has been re-established.

## A natural limit to debris flow bulking

The positive correlation between flow parameters such as volume, shear stress, and flow depth imply that there is a runaway mechanism that strongly promotes bulking during debris flow. As a debris flow erodes bed material it grows in volume, flow depth and shear stress thereby promoting even more erosion and bulking, potentially leading to orders of magnitude increases in flow volume. Indeed, such behaviour has been observed in nature and is reproduced by numerical models^14,23^. Our data, however, shows that there is a natural limit to this bulking set by channel geometry. The large flow of June 21 illustrates that when a flow exceeds the channel capacity it starts forming levee and overbank deposits. This can lead to net deposition, despite substantial thalweg erosion, thereby limiting or even counterbalancing further flow bulking. Schürch et al.^29^ previously found that in the Illgraben channel erosion becomes more likely with increased flow depth and shear stress, although a broad range of outcomes is possible at any given flow depth or shear stress. They further found that the transition from deposition to erosion typically occurs around a flow depth of 1.5 m, explaining the observed pattern of erosion of the deep channel thalweg and the simultaneous deposition on overbanks where flow depths are relatively small and do not exceed 1.5 m.

This finding implies that channel geometry may set a limit to the maximum size that a debris flow might obtain through bulking, as material will start to deposit once the flow reaches the overbanks. Because channel geometry may also vary along a stream and over time, we can expect contrasting spatio-temporal responses. These results emphasize that understanding and resolving both erosion and deposition processes and rates is of equal importance for flow bulking and volume estimates.

## Towards estimating bulking of debris flows in nature

The importance of incorporating debris-flow bulking in hazard assessments has been increasingly recognised in recent years, there has been an increasing number of studies on debris-flow erosion, and erosion equations are being incorporated into numerical models^21–23^. Our observations reveal critical but generally overlooked phenomena controlling erosion in debris flows torrents. These results call for more high-resolution spatio-temporal datasets of debris-flow erosion and deposition, stress that check dams should be taken into account for flow bulking estimation, call for further investigation into memory effects, and stress that we should not only strive to understand and predict erosion processes but should understand deposition processes as well^51^, before we can accurately estimate and model debris-flow entrainment and bulking. Where erosion is increasingly incorporated into models, deposition processes have to date been largely overlooked.

## Methods

### Point cloud generation

We have generated digital elevation models (DEMs) (5 cm ground sampling distance) and orthomosaics (2.5 cm ground sampling distance) of the channel bed with drone-based photogrammetry. We used a DJI Mavic Pro 2, with has a 1″ CMOS sensor on-board collecting raw imagery with a resolution of 20 megapixels. We collected imagery with a side overlap of 80% and a forward overlap of 70%. We took nadir images from both channel banks and the middle of the channel and images with a 25° off-nadir camera pitch over the middle of the channel looking in an upstream direction to minimize doming effects^52^. Images were obtained at an altitude of 100 m above ground level resulting in a ground sampling distance of 2.5 cm. The total survey contained 900–1,000 images. We used anthropogenic and natural terrain features as ground control points (gcps), such as manholes, road surface marks, and cobbles and boulders. Because some boulders became buried or removed over time, we used 66 gcps for the first time step in November 2018 and 51 gcps for the last time step in July 2019. Point clouds were constructed with Agisoft Metashape 1.5. Point cloud accuracy at the gcps as reported by Metashape ranged between 0.21 and 0.27 m (Suppl. Table 1), and are substantially smaller than the typical observed erosion and deposition amounts (Figs. 2, 3).

### Point cloud filtering and DEM generation

To improve accuracy of channel-bed topography we removed erroneous points and overhanging vegetation from the dense point clouds before creating the raster DEM using LAStools (rapidlasso GmbH). The filter procedure has two main steps: remove low noise and filter overhanging vegetation, but otherwise aims to retain all natural detail in the channel and avoids clipping at steep sections at the channels edge. Low noise points are typical for dense point clouds generated using UAV photogrammetry. To filter these points, we removed points more than 0.1 m below a smoothed 20th height percentile surface with at step size of 0.5 m. Overhanging vegetation was removed by classifying ground points using the lasground functionality in LAStools with ‘ultra fine’ settings. This setting only effectively removes overhanging and sparse vegetation in the channel, but retains most of the fine details in the channel at the expense of including dense vegetation in geomorphologically inactive areas which were not of interest to our analysis. Filtered points were rasterized into a DEM with a ground sampling distance of 5 cm.

### Erosion and deposition quantification

Erosion and deposition as a result of debris-flow activity was quantified as follows (Suppl. Figure 2). Initially, we generated DEMs of difference by subtracting the pre-flow DEM from the post-flow DEM. Subsequently, we used the mudlines and levees left behind by the debris flow to manually digitize the extent of each debris flow. We used this extent to clip out the DEMs of difference, such that we only consider topographic changes in the area affected by a debris flow. This method yields a conservative estimate for erosion because it includes deposition in the falling limb of the flow hydrograph^31^. In the Illgraben torrent debris flows substantially alter channel bed topography, while floods hardly affect the channel bed—their geomorphic work is generally limited to reworking of the channel bed and winnowing of fines^31^. We therefore assume that the measured changes are largely attributed to debris-flow activity. We extracted erosion volume, deposition volume, and net volume changes for segments of 10 m along channel (Suppl. Figure 2). We did exclude areas within 2 m of check dams, because tiny (~ 20 cm) offsets between the DEMs could otherwise generate large, but incorrect, erosion or deposition volumes.

## Supplementary information

Supplementary Information.

Supplementary Movie 1.

Supplementary Movie 2.

Supplementary Movie 3.

Supplementary Movie 4.
