# Supplementary material for: How memory effects, check dams, and channel geometry control erosion and deposition by debris flows
Source: Sci Rep. 2020 Aug 20;10:14024. doi: 10.1038/s41598-020-71016-8 (PMC7441160; doi:10.1038/s41598-020-71016-8)
Supplement: Supplementary file 1 — Supplementary Information. [file 41598_2020_71016_MOESM1_ESM.docx]

SUPPLEMENT TO:

How memory effects, check dams, and channel geometry control erosion and deposition by debris flows

T. de Haas^1^*, W. Nijland^1^, S. M. de Jong^1^, B. W. McArdell^2^

^1^Department of Physical Geography, Universiteit Utrecht, Utrecht, The Netherlands.

t.dehaas@uu.nl

^2^Swiss Federal Institute for Forest, Snow and Landscape Research WSL, Birmensdorf, Switzerland.

**Supplementary Tables**

Suppl. Table 1: Characteristics of the topographic surveys.

| Acquisition date | Number of pictures | Number of GCPs | RMSE of GCPs (m) |
| --- | --- | --- | --- |
| November 8, 2018 | 883 | 66 | 0.23 |
| April 30, 2019 | 1432 | 63 | 0.21 |
| June 16, 2019 | 970 | 59 | 0.27 |
| June 22, 2019 | 968 | 59 | 0.23 |
| July 4, 2019 | 940 | 51 | 0.21 |

**Supplementary Figures**

**
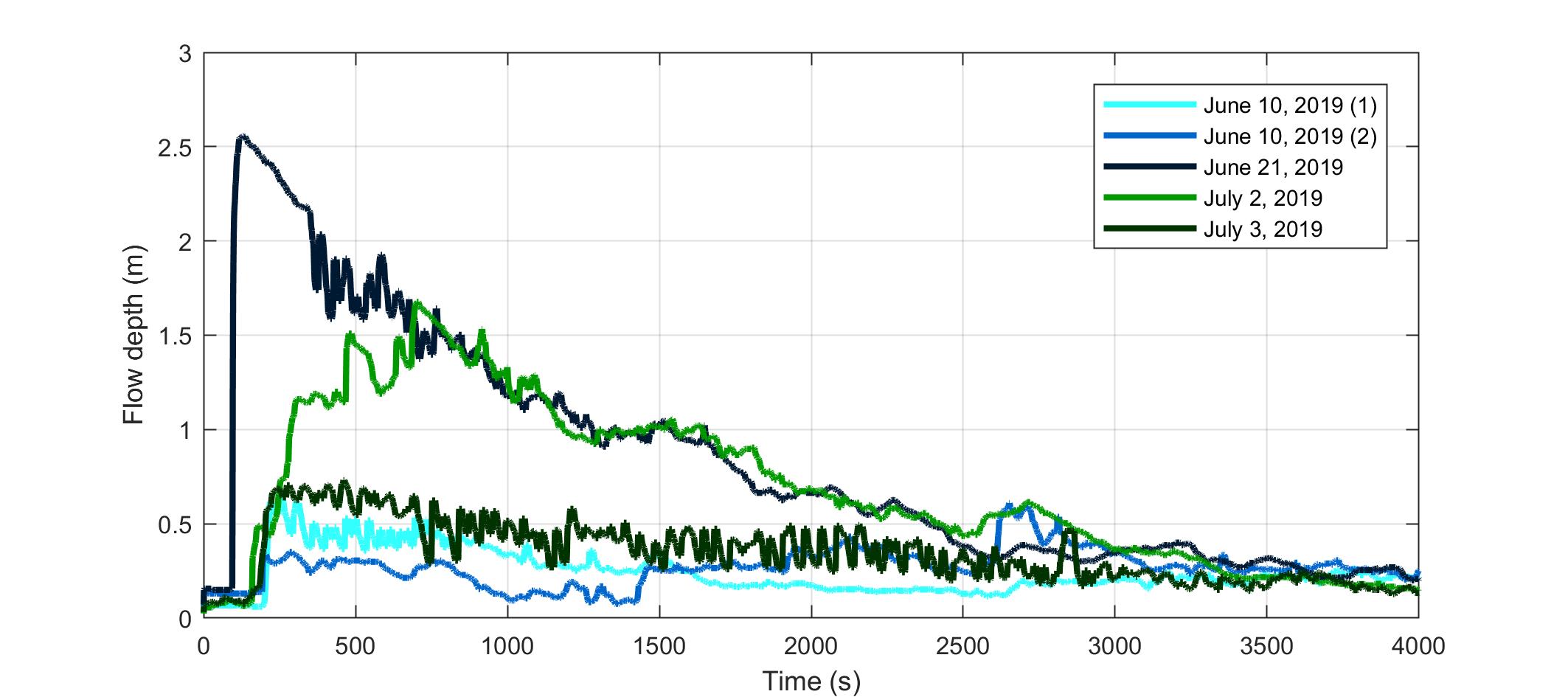
**

Suppl. Fig. 1: Hydrographs of the studied debris flows at the measurement station.

**
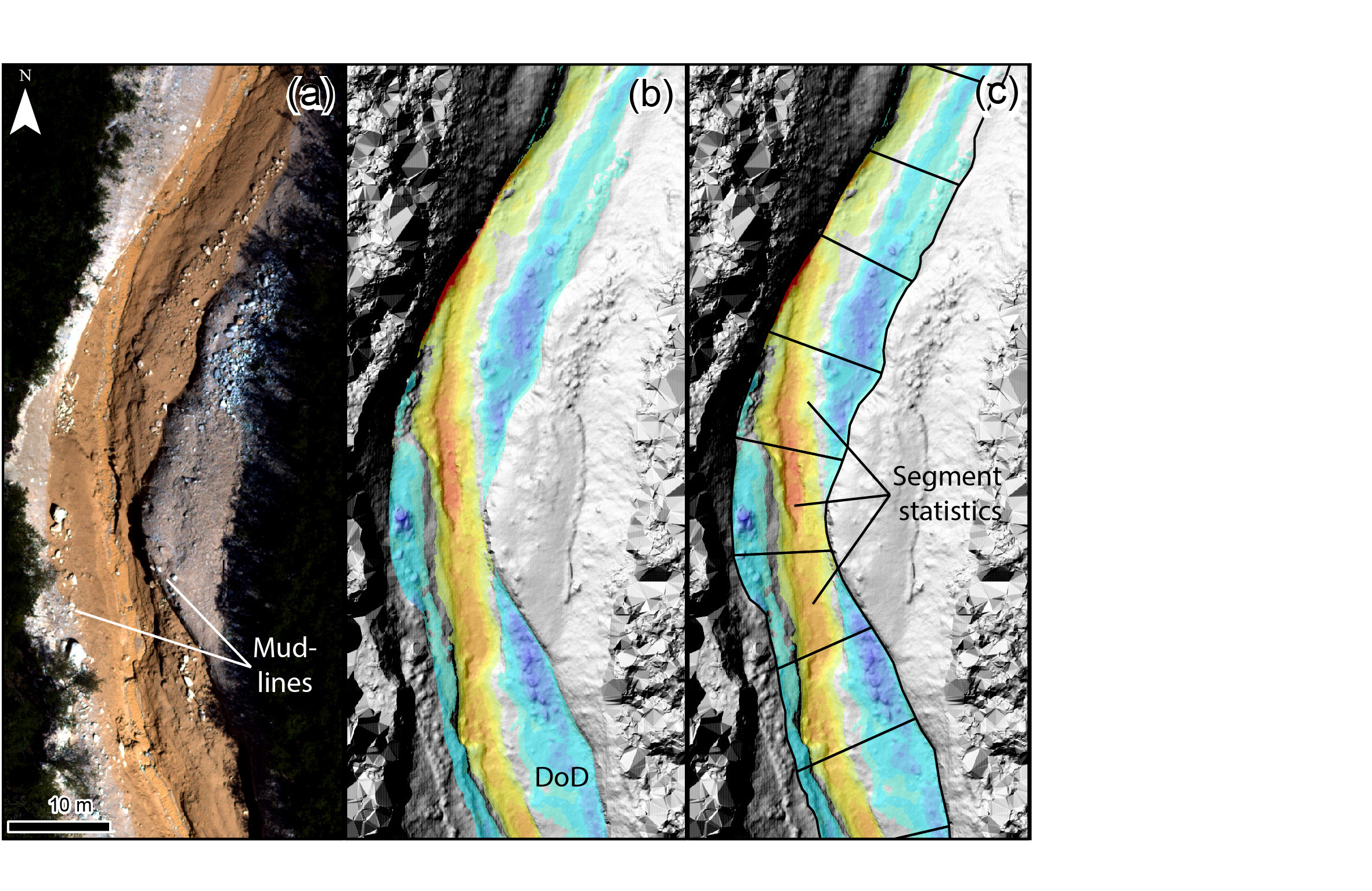
**

Suppl. Fig. 2: Illustration of the methodology to calculate erosion and deposition. (a) The darker colour of the recent flow deposit as seen on the orthomosaic was used to delineate the flow extent. (b) The flow extent was then used to calculate the elevation difference between the pre- and post-flow DEMs. (c) We created 10 m along-channel segment for which we calculated erosion volume, deposition volume, and net volume change. Flow is from bottom to top. The images show the debris flow of July 3, 2019. Panels b and c show the hillshaded DEM overlain by the DEM of difference, on which warm colours denote deposition and cold colours denote erosion*,* values ranging between -0.25 and 0.25 m are transparent. Value range from red to blue is -2 to 2 m channel-bed elevation change. Flow is from bottom to top.

**Supplementary GIF movies**

Suppl.Movie 1: Temporal overview of Fig. 3a-c.

Suppl.Movie 2: Temporal overview of Fig. 3d-f.

Suppl.Movie 3: Temporal overview of Fig. 3g-i.

Suppl.Movie 4: Temporal overview of Fig. 3j-o.
