# Supplementary figures and images for: How memory effects, check dams, and channel geometry control erosion and deposition by debris flows
Source: Sci Rep. 2020 Aug 20;10:14024. doi: 10.1038/s41598-020-71016-8 (PMC7441160; doi:10.1038/s41598-020-71016-8)

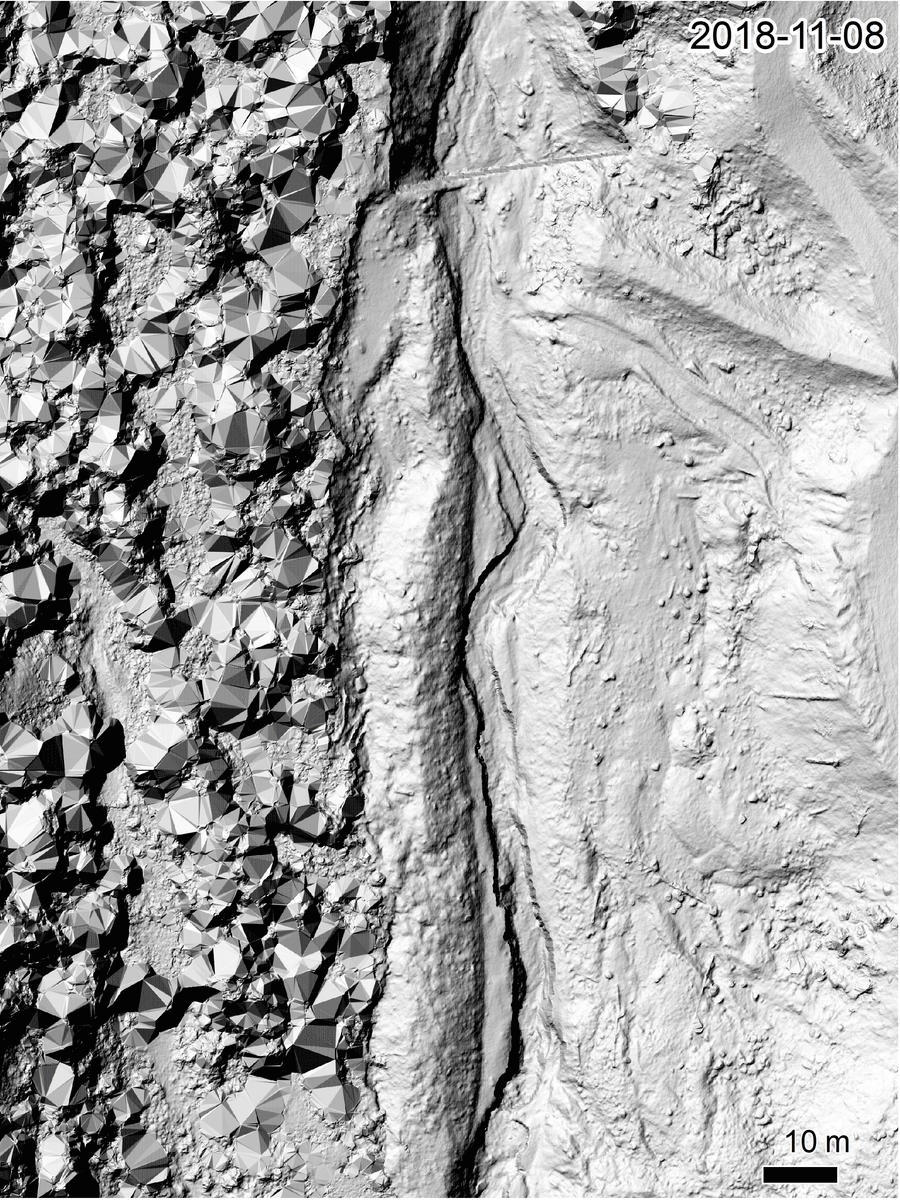

Supplement: Supplementary file 2 — Supplementary Movie 1. [file 41598_2020_71016_MOESM2_ESM.gif]

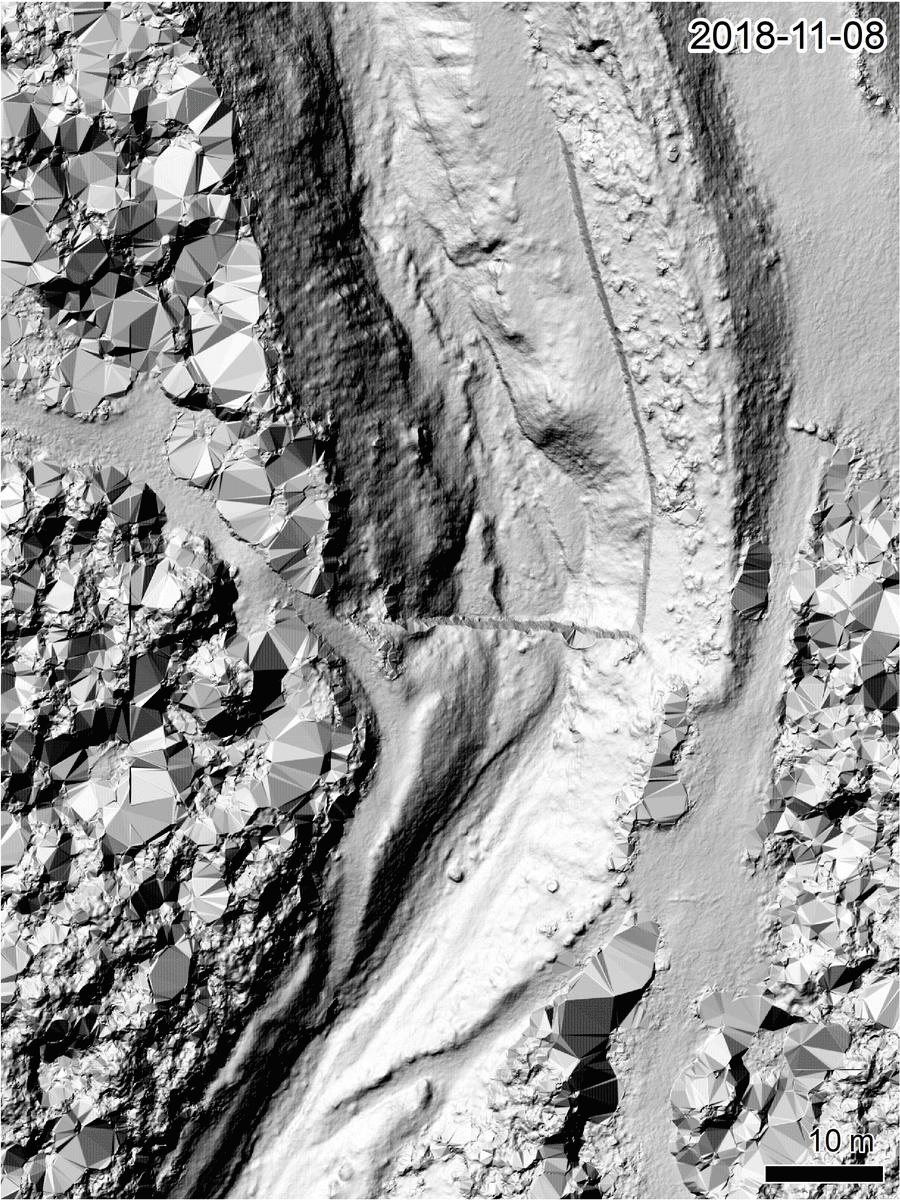

Supplement: Supplementary file 3 — Supplementary Movie 2. [file 41598_2020_71016_MOESM3_ESM.gif]

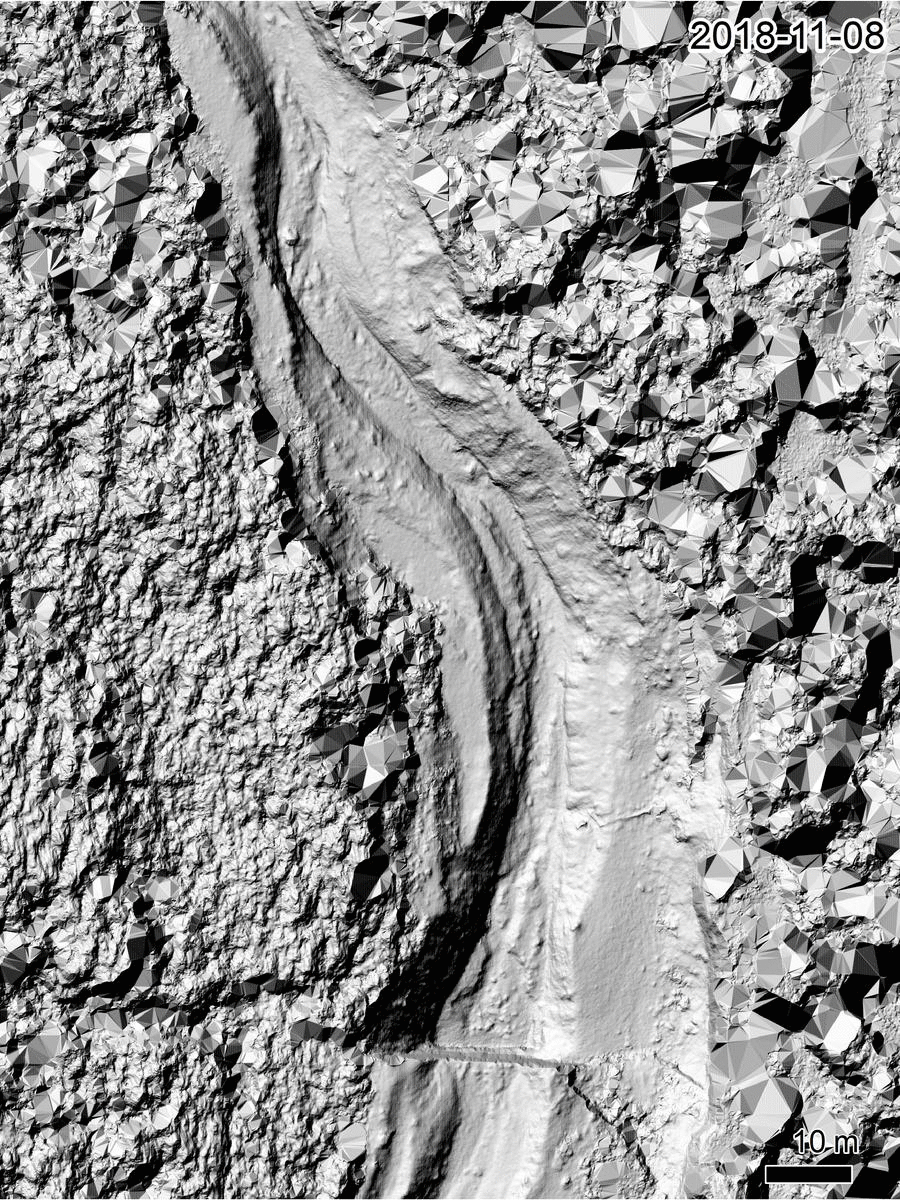

Supplement: Supplementary file 4 — Supplementary Movie 3. [file 41598_2020_71016_MOESM4_ESM.gif]

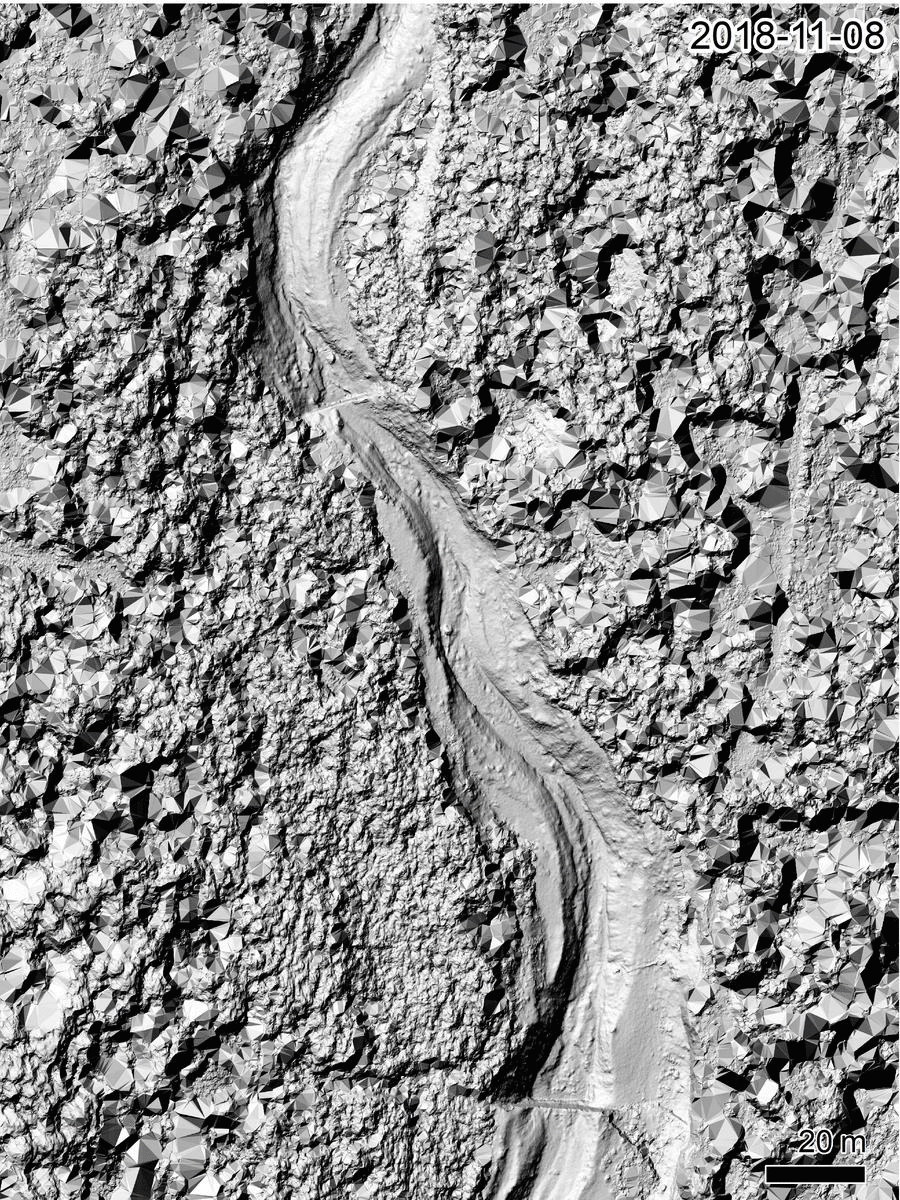

Supplement: Supplementary file 5 — Supplementary Movie 4. [file 41598_2020_71016_MOESM5_ESM.gif]
